# Identification and characterization of capsule depolymerase Dpo48 from *Acinetobacter baumannii* phage IME200

**DOI:** 10.7717/peerj.6173

**Published:** 2019-01-14

**Authors:** Yannan Liu, Zhiqiang Mi, Liyuan Mi, Yong Huang, Puyuan Li, Huiying Liu, Xin Yuan, Wenkai Niu, Ning Jiang, Changqing Bai, Zhancheng Gao

**Affiliations:** 1 Department of Respiratory and Critical Care Medicine, Peking University People’s Hospital, Beijing, China; 2 Department of Respiratory and Critical Care Medicine, 307th Hospital of PLA, Beijing, China; 3 State Key Laboratory of Pathogen and Biosecurity, Beijing Institute of Microbiology and Epidemiology, Beijing, China

**Keywords:** *Acinetobacter baumannii*, Bacteriophage, Genome, Capsule depolymerase, Antivirulence

## Abstract

**Background:**

The emergence of multidrug- or extensively drug-resistant *Acinetobacter baumannii* has made it difficult to treat and control infections caused by this bacterium. It is urgently necessary to search for alternatives to conventional antibiotics for control of severe *A. baumannii* infections. In recent years, bacteriophages and their derivatives, such as depolymerases, showed great potential as antibacterial or antivirulence agents against bacterial infections. Nonetheless, unlike broad-spectrum bactericidal antibiotics, phage-encoded depolymerase targets only a limited number of bacterial strains. Therefore, identification of novel depolymerases and evaluation of their ability to control *A. baumannii* infections is important.

**Methods:**

A bacteriophage was isolated from hospital sewage using an extensively drug-resistant *A. baumannii* strain as the host bacterium, and the phage’s plaque morphology and genomic composition were studied. A polysaccharide depolymerase (Dpo48) was expressed and identified, and the effects of pH and temperature on its activity were determined. Besides, a serum killing assay was conducted, and amino acid sequences homologous to those of putative polysaccharide depolymerases were compared.

**Results:**

Phage IME200 yielded clear plaques surrounded by enlarged halos, with polysaccharide depolymerase activity against the host bacterium. A tail fiber protein with a Pectate_lyase_3 domain was identified as Dpo48 and characterized . Dpo48 was found to degrade the capsule polysaccharide of the bacterial surface, as revealed by Alcian blue staining. Dpo48 manifested stable activity over a broad range of pH (5.0–9.0) and temperatures (20–70 °C). Results from *in vitro* serum killing assays indicated that 50% serum was sufficient to cause a five log reduction of overnight enzyme-treated bacteria, with serum complement playing an important role in these killing assays. Moreover, Dpo48 had a spectrum of activity exactly the same as its parental phage IME200, which was active against 10 out of 41 *A. baumannii* strains. Amino acid sequence alignment showed that the putative tail fiber proteins had a relatively short, highly conserved domain in their N-terminal sequences, but their amino acid sequences containing pectate lyase domains, found in the C-terminal regions, were highly diverse.

**Conclusions:**

Phage-encoded capsule depolymerases may become promising antivirulence agents for preventing and controlling *A. baumannii* infections.

## Introduction

*Acinetobacter baumannii* is an opportunistic pathogen that can cause major health problems in immunocompromised individuals, especially patients in intensive care units (Elhosseiny & Attia, 2018). The clinical manifestations of *A. baumannii* infections include ventilator-associated pneumonia, urinary tract infection, wound and soft tissue infections, meningitis, intra-abdominal infection, bacteremia, and catheter-related infection (Almani et al., 2017; Hurley, 2016; Shojaei et al., 2016; Teng et al., 2015). In recent years, the use of broad-spectrum antibiotics has increased, and many *A. baumannii* strains have been shown to develop antibiotic resistance. The overuse of antibiotics has led to the emergence of multidrug-resistant *A. baumannii*, extensively drug-resistant *A. baumannii*, and even pandrug-resistant *A. baumannii*, which cause great difficulties for clinicians in the control and treatment of *A. baumannii* infections (Ren et al., 2016; Swe Swe-Han, Mlisana & Pillay, 2017; Zheng, Yuan & Li, 2013). Therefore, alternatives to conventional antibiotics are urgently needed to control severe *A. baumannii* infections.

Bacteriophages are viruses that can specifically infect bacteria and utilize their hosts for replication. In recent years, bacteriophages were shown to be clinically functional and to have no obvious adverse effects when used to combat bacterial infections *in vitro* and *in vivo* in some clinical trials (Fish et al., 2016; Leitner et al., 2017; Schooley et al., 2017; Wright et al., 2009). Furthermore, bacteriophage derivatives, such as endolysins or depolymerases, have a great potential as antibacterial or antivirulence agents for bacterial infections (Cheng et al., 2017; Fujiki et al., 2018; Guo et al., 2017b; Lin et al., 2017; Pan et al., 2017). Phage-encoded polysaccharide depolymerases can degrade bacterial surface polysaccharides, which function as barriers on the bacterial cell surfaces. Disintegration of these polysaccharides exposes the interior of bacteria to a host immune attack (Lin et al., 2014b; Lin et al., 2017) or disrupts biofilms via degradation of the exopolysaccharide (EPS) matrix (Gutierrez et al., 2015). Research on depolymerases to target different bacteria has been previously reported (Hsieh et al., 2017; Lin et al., 2017; Negus et al., 2015), and the genomic characterization and structural basis of *A. baumannii* depolymerases have also been researched (Hernandez-Morales et al., 2018; Lai et al., 2016; Lee et al., 2017; Oliveira et al., 2017). Nevertheless, little is known regarding their antivirulence properties. Furthermore, just like phages that target specific bacteria, depolymerases are normally specific to a limited number of *A. baumannii* strains, and the activities of different enzymes vary greatly. Therefore, more depolymerases targeting different *A. baumannii* strains need to be identified and characterized. In the present study, we identified an enzyme (Dpo48) that can strip *A. baumannii* capsule polysaccharide (CPS) and determined its activity under different pH and temperature conditions. Besides, a serum sensitivity assay mediated by Dpo48 was conducted. Thus, we tested the applicability of phage-encoded capsule depolymerases as a new strategy for controlling drug-resistant *A. baumannii* infections.

## Materials and Methods

### Bacterial strains and culture conditions

A total of 41 *A. baumannii* strains were isolated from sputum samples (AB1, AB2, AB3, AB5, AB9, AB10, AB11, AB12, AB15, AB16, AB17, AB18, AB19, AB007, AB043, AB139, AB178, AB220, AB237, AB295, AB358, AB1610, AB1611, AB1612, AB1613, AB1614, AB1615, AB-E85, and LAB-9), blood samples (AB4, AB6, AB8, AB13, AB20, AB387, and AB5-2), throat swab samples (AB392 and AB406), a bronchoalveolar lavage fluid sample (AB363), a central venous catheter sample (AB7), and a wound secretion sample (AB14) from patients with severe pneumonia in the intensive care unit at PLA Hospital 307, Beijing, China. These samples were collected from different patients on different dates from 2015 to 2017, and the patients were orally informed that the samples would be used for the screening of bacteria. The supernatants of different samples were cultured on Columbia blood agar plates (Oxoid, Hampshire, UK), and each isolate was selected from a single clone for further cultivation. Identification of the above *A. baumannii* strains and determination of their susceptibility rates toward antibiotics were carried out on a Vitek 2.0 system (BioMerieux Clinical Diagnostics, Paris, France), and the minimum inhibitory concentrations (MICs) were determined in accordance with the guidelines of the Clinical and Laboratory Standards Institute (CLSI). *Pseudomonas aeruginosa* ATCC 27853 served as a control strain. These *A. baumannii* strains were next verified by 16S ribosomal rRNA gene sequencing. The susceptibility rates of all *A. baumannii* strains toward different classes of antibiotics are given in Table 1, and all strains manifested resistance to at least three classes of antimicrobial agents, except for five *A. baumannii* strains (AB19, AB220, AB237, AB295, and LAB-9). All the bacterial strains were stored in Luria-Bertani (LB) broth (Oxoid) with 50% glycerol (v/v) at −70 °C, and single colonies were cultured in LB broth at 37 °C, with shaking at 220 rpm. The protocols for collecting clinical samples and screening of bacteria were approved by the Ethics Committees of PLA Hospital 307 and the Beijing Institute of Microbiology and Epidemiology (Ethical Application Ref: ky-2015-3-17).

**Table 1:** Susceptibility rates toward different classes of antibiotics for clinical strains of *A. baumannii*.

**Antimicrobial category**	**Antimicrobial agent**	**Susceptible (%)**	**Intermediate (%)**	**Resistant (%)**
Penicillins +*β*-lactamase inhibitors	Ampicillin-sulbactam	14.63 (6/41)	7.32 (3/41)	78.05 (32/41)
Antipseudomonal penicillins +*β*-lactamase inhibitors	Piperacillin-tazobactam	14.63 (6/41)	12.20 (5/41)	73.17 (30/41)
Extended-spectrum cephalosporins	Cefepime	12.20 (5/41)	4.88 (2/41)	82.92 (34/41)
Ceftazidime	4.88 (2/41)	14.63 (6/41)	80.49 (33/41)
Ceftriaxone	0	14.63 (6/41)	85.37 (35/41)
Antipseudomonal carbapenems	Imipenem	17.07 (7/41)	0	82.93 (34/41)
Meropenem	17.07 (7/41)	0	82.93 (34/41)
Aminoglycosides	Amikacin	63.41 (26/41)	4.88 (2/41)	31.71 (13/41)
Gentamicin	19.51 (8/41)	0	80.49 (33/41)
Tobramycin	21.95 (9/41)	0	78.05 (32/41)
Folate pathway inhibitors	Trimethoprim-sulphamethoxazole	36.59 (15/41)	0	63.41 (26/41)
Antipseudomonal fluoroquinolones	Ciprofloxacin	12.20 (5/41)	0	87.80 (36/41)
Levofloxacin	12.20 (5/41)	21.95 (9/41)	65.85 (27/41)

### Bacteriophage isolation, purification, and plaque characterization

Among all the clinical strains, *A. baumannii* AB1610 was resistant to all classes of antibiotics, including polymyxins and tetracyclines (Table S1); thus, it could be designated as extensively drug-resistant *A. baumannii* (Magiorakos et al., 2012; Sweeney et al., 2018). Therefore, bacteriophage IME200 was isolated from raw sewage collected at PLA Hospital 307, using *A. baumannii* AB1610 as the host bacterium. Briefly, sewage samples were centrifuged at 8,000 × *g* for 10 min, and the supernatants were collected and passed through a membrane filter with 0.20 µm pore size to remove remaining bacterial debris. To obtain the target phage, 4 mL of filtered sewage and 200 µL of bacterial culture in the exponential growth phase (optical density at 600 nm [OD_600_] = 0.6) were added to 2 mL of a 3 × LB medium at 37 °C for overnight incubation with shaking at 220 rpm. The cultures containing the phage were rid of remaining bacterial cells and stored at 4 °C. The phage was isolated and purified with successive single-plaque assays using double-layer agar plates (Kropinski et al., 2009). To further characterize phage plaques, the double-layer agar plate assay was conducted. Briefly, 100 µL of the purified phage was diluted and combined with 200 µL of overnight-cultured bacteria. The mixture was then added to 4 mL of molten soft nutrient agar (0.75% agar) and rapidly overlaid onto 1.5% agar plates. The plates were incubated at 37 °C after the top layer of soft agar solidified, and phage plaques were examined at 12, 24, 48, and 72 h.

### Multilocus sequence typing (MLST) of *A. baumannii* strains and determination of the lytic spectrum of phage IME200

All strains of *A. baumannii* isolated from clinical samples were typed by the MLST method according to the protocol described for *A. baumannii* strains on the MLST website (https://pubmlst.org/abaumannii/) (Bartual et al., 2005). Sequences of seven housekeeping genes (*gltA*, *gyrB*, *gdhB*, *recA*, *cpn60*, *gpi*, and *rpoD*) were aligned using the online National Center for Biotechnology Information (NCBI) Basic Local Alignment Search Tool (BLAST), and the aligned sequences were then retrieved by comparing them to allele profiles in the *A. baumannii* MLST (Oxford) database.

The lytic spectrum of phage IME200 was determined in double-layer agar plate assays, with each of the 41 *A. baumannii* clinical isolates. Plates containing mixtures of the phage and an *A. baumannii* strain were incubated for 6 h at 37 °C, and plaque-forming units (PFU) were counted for each combination. Relative efficiency of plating (EOP) was calculated as the average PFU number of the phage on target bacteria divided by the average PFU number on host bacteria (Khan Mirzaei & Nilsson, 2015). The experiment was repeated three times.

### DNA extraction, whole-genome sequencing, and bioinformatic analysis

DNase I (Thermo Scientific, Waltham, MA, USA) and RNase A (Thermo Scientific) were added to the phage IME200 to a final concentration of 1 µg/mL each and incubated overnight at 37 °C to remove host nucleic acids. The mixture was then inactivated for 15 min at 80 °C. Ethylenediamine tetraacetic acid (EDTA; 200 µg/mL; Thermo Scientific), proteinase K (50 µg/mL; Invitrogen, Carlsbad, CA, USA), and sodium dodecyl sulfate (SDS; 0.5 mg/mL; Invitrogen) were added to the mixture, which was then incubated for 1 h at 56 °C. After that, an equal volume of saturated phenol (pH 7.9; Thermo Scientific) was added, followed by gentle vortexing and centrifugation at 10,000× g for 5 min to separate the upper aqueous phase. Equal volumes of phenol: chloroform: isoamyl alcohol (25:24:1; Sigma-Aldrich, St. Louis, MO, USA) and chloroform (Hushi, Shanghai, China) were next added as described above to purify the phage. Finally, an equal volume of isopropanol (Hushi) was added to the DNA, and the mixture was incubated at −20 °C for at least 1 h and centrifuged at 10,000× g for 10 min, and the precipitate was washed with 75% ethanol (Hushi). The genomic DNA of the phage was dissolved in deionized water and stored at −20 °C.

The genomic DNA of phage IME200 was subjected to high-throughput sequencing on a Life Technologies Ion Personal Genome System (San Francisco, CA, USA). The complete genome sequence of the phage was assembled in Newbler 2.9.1 software (Roche, Indianapolis, IN, USA), and annotated by the Rapid Annotation in Subsystem Technology (RAST; http://rast.nmpdr.org/). The putative function of the protein was predicted using NCBI BLASTP, and tRNAs were predicted in tRNAScan-SE and ARAGORN (Laslett & Canback, 2004; Schattner, Brooks & Lowe, 2005). Homology analysis of the phage genome was performed via NCBI BLAST, and the whole-genome comparison was performed by means of the BLASTN program in local BLAST-2.2.31+. After the highly homologous sequences of phage genomes were analyzed and compared, a comparison of their amino acid sequences that are encoded by putative tail fiber genes was performed in CLC Genomics Workbench 3.6.1 (CLC bio, Aarhus, Denmark). The whole genome sequence of phage IME200 is available from GenBank/EMBL/DDBJ under accession number KT804908.2.

### Expression, purification, and identification of Dpo48

Based on the annotation results of the complete genome sequences, the phage tail fiber protein gene (open reading frame 48 [ORF48]; GenBank/EMBL/DDBJ accession number ALJ97635) with a Pectate_lyase_3 domain in the C-terminal sequence was predicted to encode a putative polysaccharide depolymerase (Dpo48). The sequence of ORF48 was PCR-amplified with forward and reverse primers (5′-ATGAATATACTACGCTCATTTACAG-3′ and 3′-TTAAAATCCAGATACCACAGTAAA CATAGC-5′) carrying restriction endonuclease sites *Bam* HI (New England Biolabs [NEB], MA, USA) and *Xho* I (NEB), respectively. The amplified target product was cloned into the prokaryotic expression vector pET28a (Novagen, Madison, WI, USA) with a C-terminal His ×6 tag. The recombinant plasmid was verified by DNA sequencing on a 3730XL system (Thermo Scientific) and transfected into *E. coli* BL21 (DE3; TransGen Biotech, Beijing, China). The cells carrying recombinant plasmids were selected on LB agar plates containing 1 µg/mL kanamycin (Sigma-Aldrich). The recombinant isolates were cultured to the exponential growth phase, and then induced with 23.83 mg/mL isopropyl β-D-1-thiogalactopyranoside (IPTG; Sigma-Aldrich). After overnight culturing, the cells were centrifuged at 13,000× g for 10 min at 4 °C, resuspended in lysis buffer (50 mM NaH_2_PO_4_, 300 mM NaCl, pH 8.0), and sonicated on ice (8–10 cycles with 30 s pulse and 30 s pause). The bacterial lysate was then centrifuged at 13,000× g for 10 min at 4 °C, and the supernatant was passed through a 0.22 µm filter. The filtrate was loaded onto a Ni-NTA column (Sangon Biotech, Shanghai, China), and proteins were eluted with 5 volumes of imidazole-containing buffer (50 mM NaH_2_PO_4_, 300 mM NaCl, 250 mM imidazole, pH 8.0) via a step gradient to recover the purified Dpo48. The latter was then dialyzed overnight in a bag of 8–14 kDa molecular-mass-cutoff membrane (Viskase, Willowbrook, IL, USA), against lysis buffer at 4 °C. The molecular weight of Dpo48 was determined by sodium dodecyl sulfate 10% polyacrylamide gel electrophoresis (SDS-PAGE; Thermo Scientific) followed by staining with Coomassie blue (Sigma-Aldrich). Dpo48 was then quantified with the Bradford Protein Assay Kit (Thermo Fisher Scientific).

The depolymerase activity of Dpo48 was qualitatively assayed by modified single-spot assays. In brief, 200 µL of overnight bacterial culture was added to 4 mL of molten soft nutrient agar and incubated at 37 °C for 3 h to form a bacterial lawn in plates. The purified enzyme (0.5 mg/mL) was serially diluted, then 4 µL of each dilution was dropped onto an AB1610 bacterial lawn for incubation at 37 °C overnight. The plates were monitored for the formation of semiclear spots as a measure of enzymatic activity. In addition, the sensitivity of other *A. baumannii* strains to Dpo48 (0.25 µg/mL) was determined in modified single-spot assays.

### Extraction of bacterial surface polysaccharides

The extraction and purification of bacterial EPS were performed via previously described methods (Hsieh et al., 2017) with some minor modifications. Briefly, clones of AB1610 were cultured overnight in the LB liquid medium with 0.25% glucose; then, 1 mL of the culture was centrifuged at 10,000× g for 5 min, and the pellet was resuspended in 200 µL of water. An equal volume of water-saturated phenol (pH 6.6; Thermo Scientific) was added, and the mixture was vortexed vigorously. The mixture was then incubated at 65 °C for 20 min, followed by chloroform extraction and centrifugation to remove bacterial debris. The extracted EPS was lyophilized overnight and stored at −20 °C.

### Determination of Dpo48 activity and Alcian blue staining

Dpo48 activity was determined by the quantification of reducing sugars released from bacterial surface polysaccharides during a reaction with 3, 5-dinitrosalicylic acid (DNS) (Oliveira et al., 2017). Briefly, the lyophilized EPS powder was resuspended in PBS to a final concentration of 2 mg/mL, and incubated with 10 µg/mL of active or inactivated (by heating at 100 °C for 15 min) Dpo48 to a final reaction volume of 1.0 mL at 37 °C for 1 h. EPS or Dpo48 alone served as controls. Two volumes of the DNS reagent (Solarbio, Beijing, China) was immediately added to each reaction mixture, which was then boiled for 5 min. Absorbance was measured at 540 nm, and the experiment was repeated three times. Data are presented as means ± standard deviation (SD).

Each sample was separated by SDS-PAGE in a 10% gel, followed by staining with Alcian blue as described elsewhere (Karlyshev & Wren, 2001; Pan et al., 2013). In brief, the gel was washed three times (5, 10, and 15 min) with the fix/wash solution (25% ethanol, 10% acetic acid in water), and then soaked in 0.1% Alcian blue (Sigma-Aldrich) dissolved in the fix/wash solution for 15 min in the dark. The gel was finally destained overnight at room temperature in the fix/wash solution. Because the EPS was covalently bound to the cell surface as CPS (Roach & Donovan, 2015), the CPS stained blue.

### Effects of pH and temperature on Dpo48 activity

The enzymatic activity at different pH levels or temperatures was assayed by methods from other studies (Majkowska-Skrobek et al., 2016), with some minor modifications. Briefly, the lyophilized extract powder was resuspended in 50 mM sodium acetate buffer (pH 3.0–5.0), 50 mM Na_2_HPO_4_ buffer (pH 6.0–7.0), or 50 mM Tris-HCl buffer (pH 8.0–9.0) to a final concentration of 2 mg/mL and incubated with 10 µg/mL active Dpo48 for 1 h at 37 °C. The final reaction volume was 1.0 mL. In addition, the polysaccharide was dissolved in a buffer with optimal pH, and incubated with Dpo48 at 20, 37, 50, 60, 70, or 80 °C for 1 h, followed by cooling to room temperature. EPS or Dpo48 alone in optimal-pH buffer incubated at 37 °C served as a control. The Dpo48 activity was then immediately measured as the reducing sugars produced, as described above. All the experiments were repeated at least three times, and the data were presented as means ± SD.

### Serum sensitivity assay

To determine the optimal volume ratio of serum to enzyme-treated bacteria in a killing assay, an experiment was conducted as previously described with some minor modifications (Pan et al., 2015). Briefly, a total of ∼10^9^ CFU/mL of overnight culture AB1610 was incubated with or without 10 µg/mL Dpo48 at 37 °C for 1 h. Approximately 10^7^ CFUs of enzyme-treated bacteria were then added to human serum from healthy donors at volume ratios of 3:1, 1:1, or 1:3 to a final reaction volume of 100 µL. After the samples were incubated for 3 h at 37 °C, the bacteria were counted in serial 10-fold dilutions on LB agar plates. The result was expressed as the CFUs of bacterial reduction, which was calculated as the initial inoculum minus viable counts. To evaluate the role of serum complement in the serum killing assay, human serum was heated to 56 °C for 30 min to inactive the serum complement. Approximately 10^7^ CFUs of enzyme-treated bacteria were added to active or inactive serum at a volume ratio of 1:1 and cultured for 3 h at 37 °C. The untreated bacteria were incubated with Dpo48 or serum as a control, and the result was expressed as the counts of bacterial reduction. All the experiments were conducted independently three times, and the results were presented as means ± SD.

### Statistical analysis

All experimental data are represented as means ± SD. The independent Student’s *t* test was utilized to compare two groups, and the one-way analysis of variance (ANOVA) was used to compare multiple groups. All statistical analyses were performed, and the results plotted in Prism 7 (GraphPad Software, La Jolla, CA, USA). Data with *P* < 0.05 were considered statistically significant.

## Results

### Bacteriophage isolation and plaque formation

**Figure 1:**
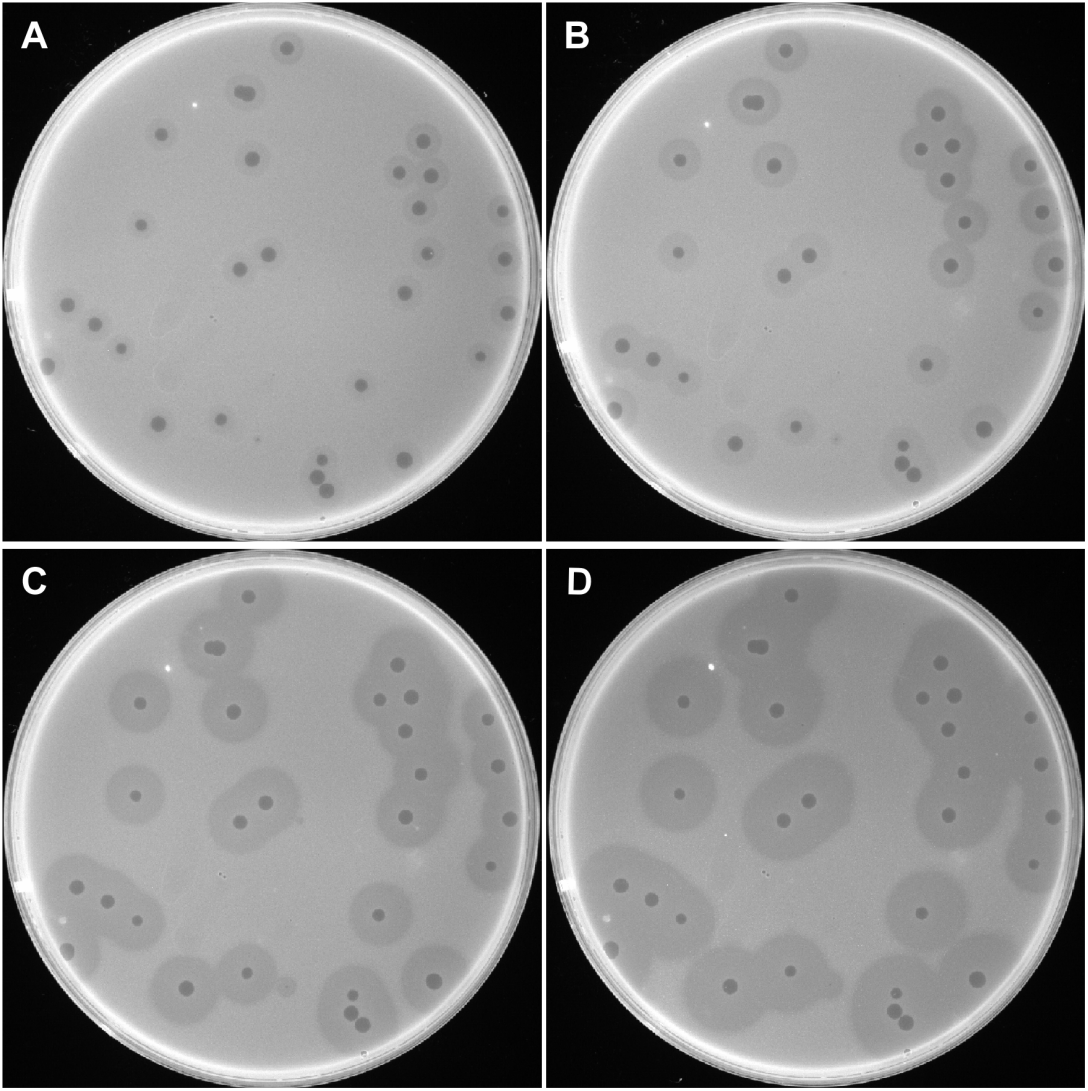
Plaques induced by phage IME200. The host bacterium *A. baumannii* AB1610 was infected with phage IME200, and the mixture was embedded in 0.75% agar and incubated on solid agar plates at 37 °C. Phage-induced plaques were observed and recorded at 12, 24, 48, and 72 h (A, B, C, and D, respectively) after infection.

A bacteriophage was isolated from raw sewage collected at PLA Hospital 307 and was designated as IME200. When plated on *A. baumannii* AB1610, the isolated phage formed small, round, clear plaques on double-layer agar plates. The plaques were surrounded by a translucent halo, which increased in diameter over time (Fig. 1). The clear plaques were ∼2 mm in diameter, while the translucent halos were ∼6, 8, 10, and 12 mm at 12, 24, 48, and 72 h, respectively. The growing halos were assumed to result from the activity of depolymerase, an enzyme that can degrade bacterial surface polysaccharides (Pires et al., 2016).

### MLST of *A. baumannii* strains and the lytic spectrum of phage IME200

Because phages are highly specific to their target bacteria, it is necessary to evaluate the lytic range of a phage before it can be used to combat bacterial infections. In a double-layer agar plate assay, the lytic spectrum of phage IME200 was tested against a panel of 41 *A. baumannii* strains, including the host bacterium. Results of the MLST analysis revealed that the 41 *A. baumannii* strains included 14 sequence types, whereas five other strains were untypeable (Table 2). Strains infected by phage IME200 belonged to ST 208 (AB5-2, AB1610, AB1611, AB1614, AB1615, AB-E85), 238 (AB4, AB5), 420 (AB220) and 1106 (LAB-9), and efficiency of plating (EOP) was greater than 0.25 (Table 2). Moreover, those 10 *A. baumannii* strains were also sensitive to Dpo48, which had the same spectrum of activity as did phage IME200.

**Table 2:** The list of *A. baumannii* strains used in MLST and their typing results, including sensitivity to phage IME200 and its depolymerase Dpo48.

**MLST type**	**Bacteria isolates**	**Sample origin**	**Sensitivity to phage**^a^	**Sensitivity to depolymerase**^b^
195	AB7	CVC	−	−
AB363	BALF	−	−
AB178, AB358	sputum	−	−
208	AB5-2	blood	+	+
AB1, AB2, AB3, AB10, AB11, AB139	sputum	−	−
AB1610, AB1611, AB1614, AB1615, AB-E85	sputum	+	+
218	AB6, AB8	blood	−	−
AB9, AB043	sputum	−	−
238	AB4	blood	+	+
AB5	sputum	+	+
280	AB237	sputum	−	−
325	AB295	sputum	−	−
368	AB18, AB007	sputum	−	−
369	AB14	wound secretion	−	−
AB387	blood	−	−
AB406	throat swab	−	−
420	AB220	sputum	+	+
451	AB13, AB20	blood	−	−
469	AB12	sputum	−	−
485	AB392	throat swab	−	−
1106	LAB-9	sputum	+	+
1214	AB1612	sputum	−	−
NT	AB15, AB16, AB17, AB19, AB1613	sputum	−	−

**Notes.**

a The efficiency of plating (EOP) was greater than 0.25.

b The concentration of Dpo48 was 0.25 μg/mL.

BALF bronchoalveolar lavage fluid CVC central venous catheter NT not typeable “−” nonsensitive “+” sensitive

### The putative tail fiber protein (ORF48) has depolymerase activity

**Figure 2:**
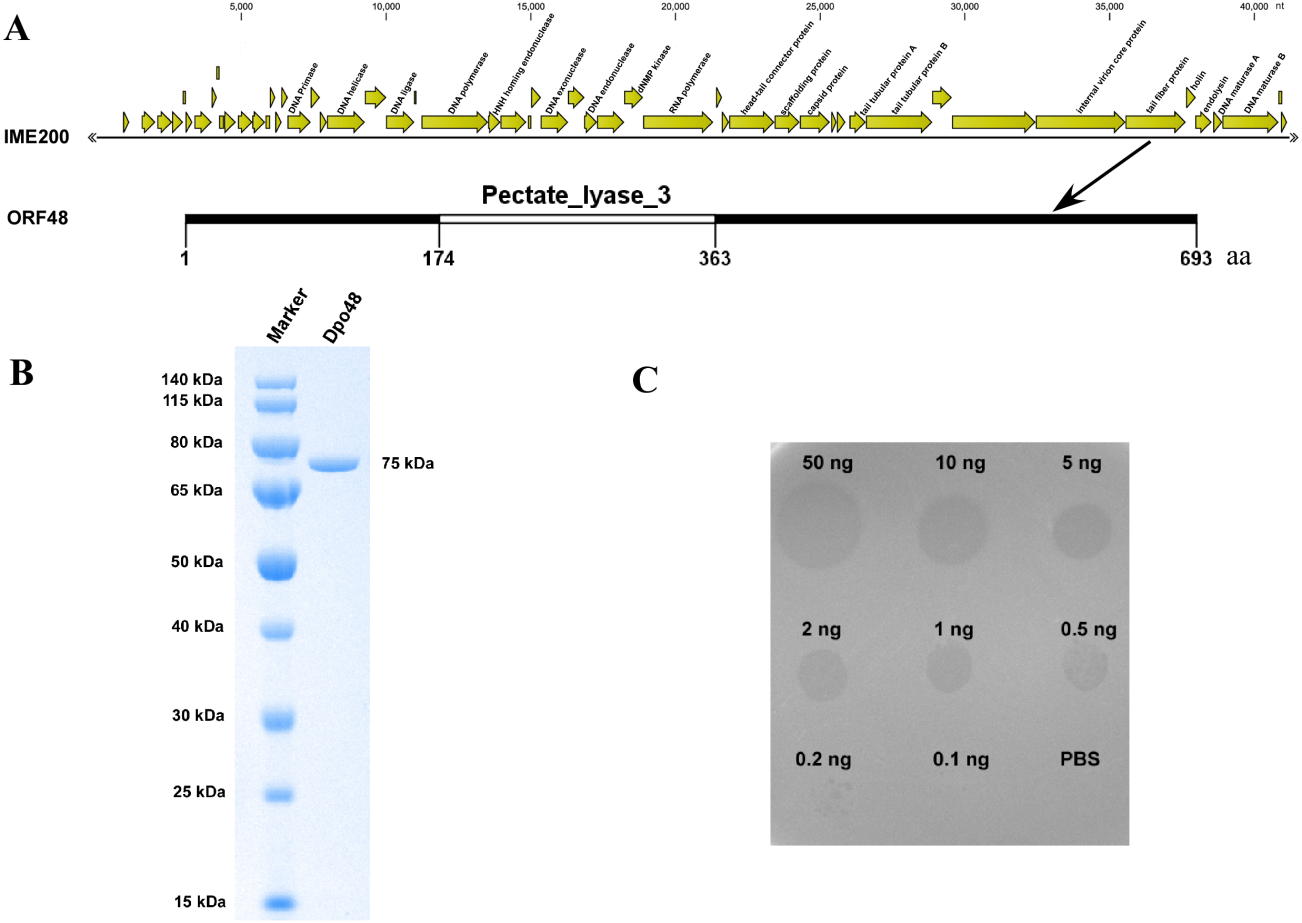
Bioinformatic analysis and expression of ORF48 and determination of its depolymerase activity. (A) The annotation and location of ORF48 with a Pectate_lyase_3 domain in the complete genome of phage IME200. (B) Expressed His-tagged recombinant Dpo48 was purified on a Ni-column and then analyzed by SDS-PAGE in a 10% gel. (C) The depolymerase activity of Dpo48 was determined by modified single-spot assays using serial dilutions of the enzyme on a lawn of the host bacterium AB1610. PBS served as a negative control.

The assembly results indicated that the genome of phage IME200 is 41,243 bp, with G+C content of 39.3%, and no tRNA genes were found. The annotation results from RAST indicated that the complete genome of phage IME200 contains 54 putative ORFs, 20 of which were predicted by NCBI BLASTP to encode functional proteins (Fig. 2A and Table S2). As also depicted in Fig. 2A, ORF48 was predicted to contain a Pectate_lyase_3 domain in the C-terminal region, which is commonly recognized as a polysaccharide depolymerase, with a predicted length and molecular weight of 693 aa and 75.141 kDa, respectively. To verify whether ORF48 has depolymerase activity, the coding sequence of the gene was cloned into a prokaryotic expression vector. As predicted, the purified expressed protein, designated as Dpo48, was found to be 75 kDa as revealed by SDS-PAGE in the 10% gel (Fig. 2B), and the concentration of purified Dpo48 turned out to be 0.5 mg/mL. A single-spot assay using the purified Dpo48 revealed that ORF48 had polysaccharide depolymerase activity and could cause a translucent halo on the lawn of the host bacteria. The size of the semiclear circles correlated with Dpo48 concentration, with the halo disappearing when the enzyme was diluted to 0.1 ng (Fig. 2C).

**Figure 3:**
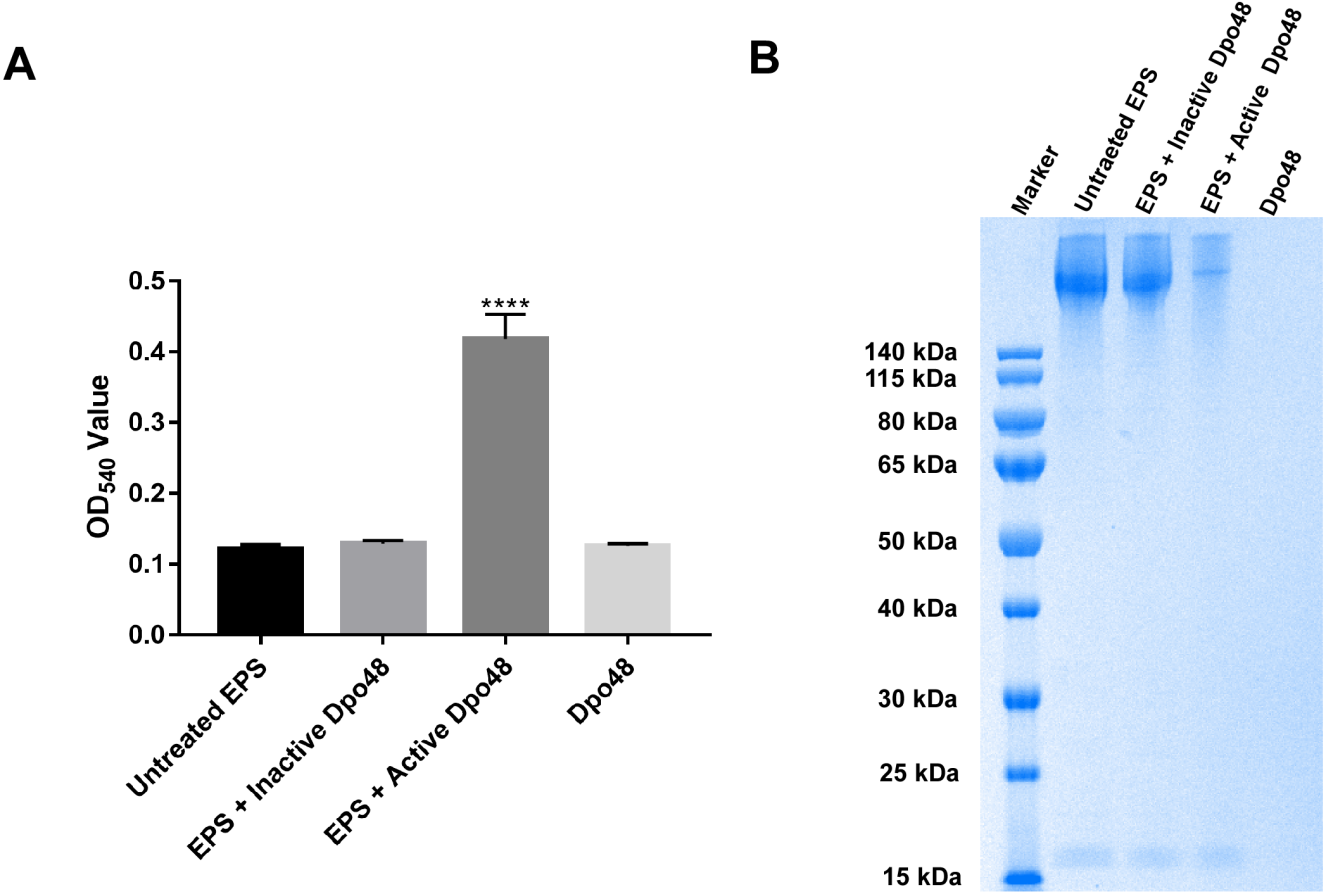
Bacterial surface polysaccharides were degraded by Dpo48. Bacterial EPS was incubated with active or inactivated Dpo48 (100 °C for 15 min) at 37 °C for 1 h, and EPS or Dpo48 alone served as controls. (A) Production of reducing sugars released from EPS was quantified at 540 nm via the reaction with DNS. Data are expressed as means ± SD (*n* = 3), and the statistical analysis was conducted by one-way ANOVA (****P < 0.0001). (B) Each sample was analyzed by SDS-PAGE in a 10% gel, and the CPS was visualized with Alcian blue staining.

The Dpo48 activity was quantified as the reducing sugars released from bacterial surface polysaccharides. As presented in Fig. 3A, OD_540_ of EPS only and of Dpo48 in PBS was 0.122 ± 0.006 and 0.126 ± 0.003, respectively. OD_540_ of EPS treated with active Dpo48 was 0.419 ± 0.035. By contrast, when the polysaccharide extracts were incubated with inactive Dpo48, OD_540_ decreased to 0.129 ± 0.005. The increased OD_540_ of the EPS treated with Dpo48, resulting from the production of reducing sugars, meant that the surface polysaccharides extracted from the host bacteria could be degraded by the depolymerase. In addition, the bacterial-capsule–degrading properties of Dpo48 were validated by gel electrophoresis followed by Alcian blue staining (Fig. 3B). Results of the gel electrophoresis also revealed that EPS could apparently be degraded by Dpo48, as compared with EPS alone, or the mixture of EPS and inactivated Dpo48, or Dpo48 alone, which failed to stain with Alcian blue. Dpo48 could therefore degrade the CPS of the bacterial surface.

### The influence of pH and temperature on the activity of Dpo48

**Figure 4:**
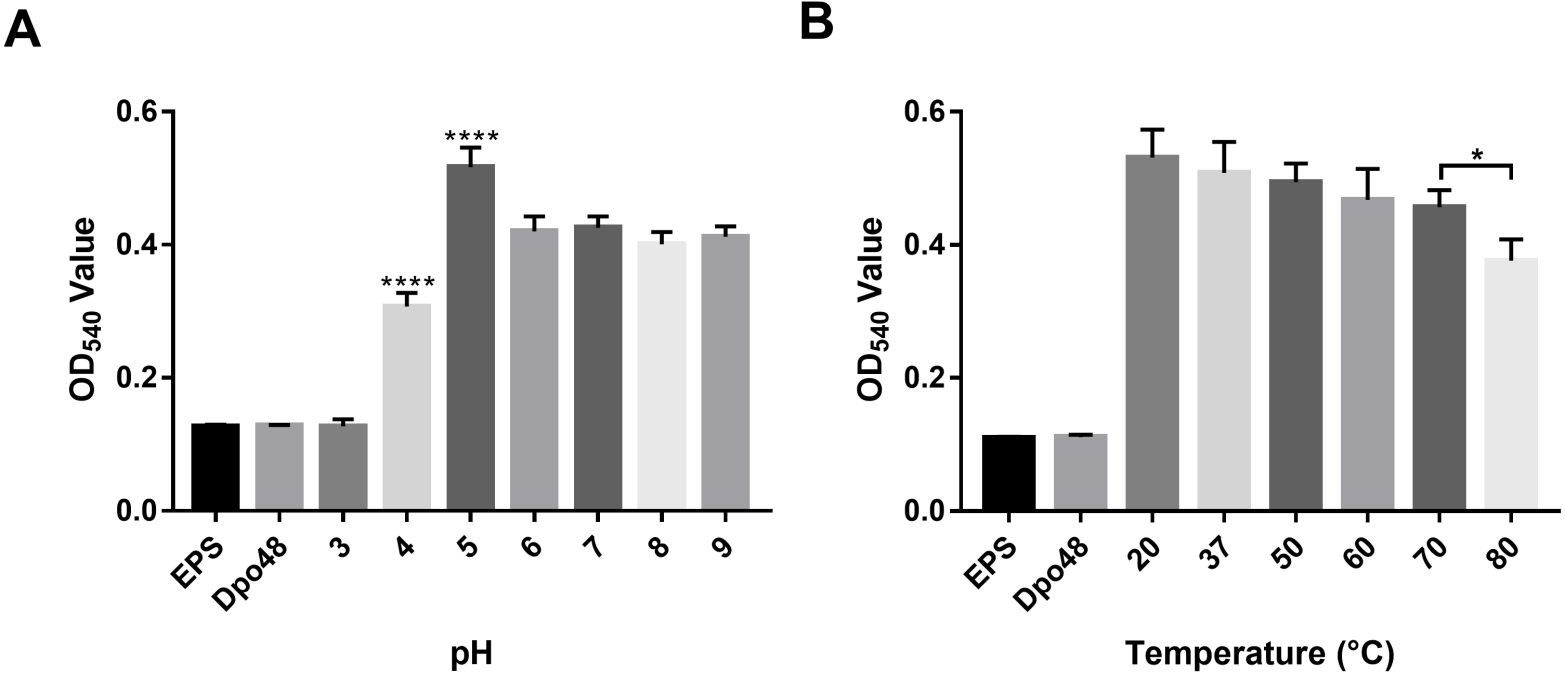
Activity of Dpo48 across a range of pH and temperatures. (A) The influence of pH on the activity of Dpo48. EPS was incubated with active Dpo48 in 50 mM sodium acetate buffer (pH 3.0–5.0), 50 mM Na_2_HPO_4_ buffer (pH 6.0–7.0), or 50 mM Tris-HCl buffer (pH 8.0–9.0) for 1 h at 37 °C. (B) The effect of temperature on Dpo48 activity. EPS at optimal pH was incubated with Dpo48 at 20, 37, 50, 60, 70, or 80 °C for 1 h. EPS or Dpo48 alone at optimal pH incubated at 37 °C served as controls. Dpo48 activity was quantified as the reducing sugars produced. The values are presented as means ± SD (*n* = 4), and the statistical analysis was assayed by one-way ANOVA. (*P < 0.05, ****P < 0.0001).

The activity of Dpo48 at different pH levels was determined by quantifying the production of reducing sugars (Fig. 4A). Results on OD_540_ of EPS treated with Dpo48 in buffers with different pH levels indicated that Dpo48 remained active in buffers ranging from pH 5.0 to 9.0, with the optimal pH being 5.0 (one-way ANOVA, *P* < 0.0001). The activity of Dpo48 diminished when pH was 4.0 and almost disappeared compared with that of EPS alone or enzyme alone when pH was 3.0 (one-way ANOVA, *P* < 0.0001). Next, the activity of Dpo48 in sodium acetate buffer (pH 5.0), at temperatures ranging from 20 to 80 °C, was determined (Fig. 4B). OD_540_ indicated that Dpo48 could maintain optimal activity at temperatures ranging from 20 to 70 °C, and that enzymatic activity diminished at 80 °C (one-way ANOVA, *P* < 0.05).

### Serum killing of the Dpo48-treated bacteria

**Figure 5:**
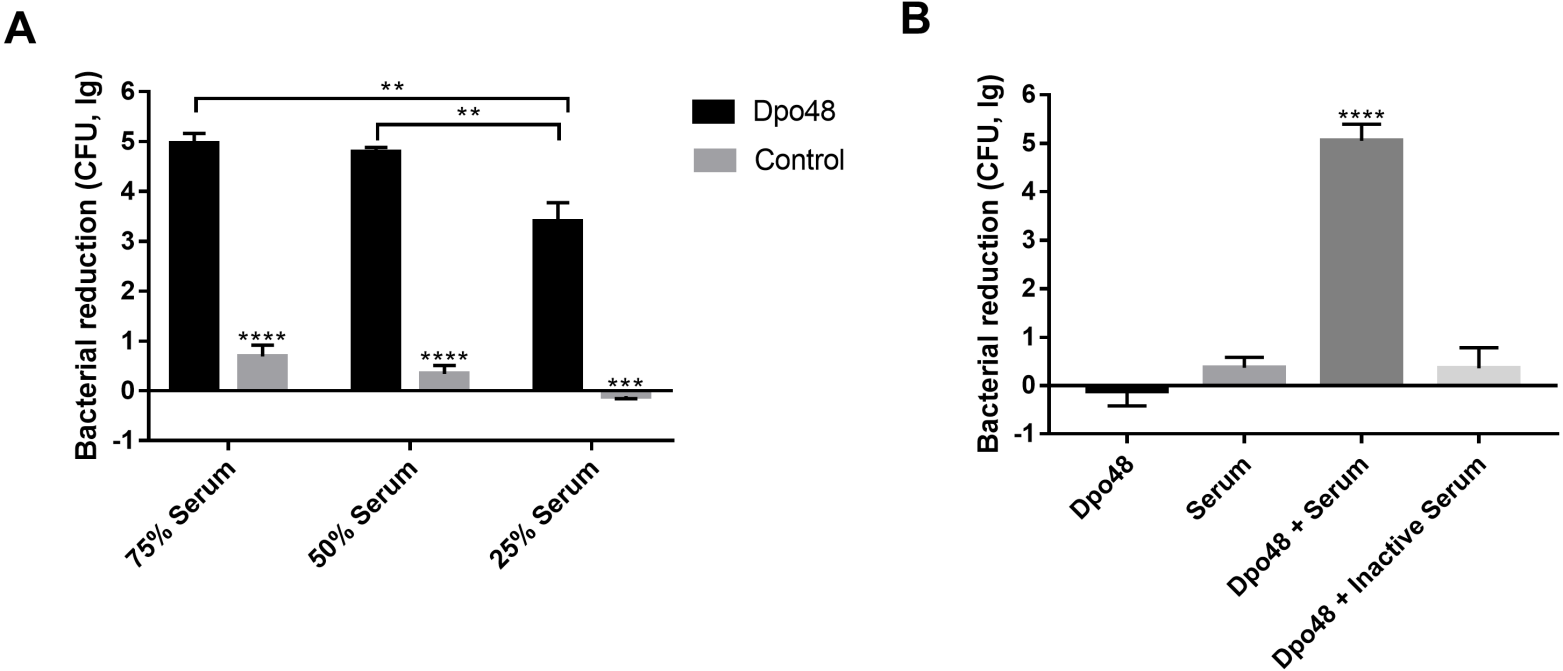
Dpo48 enhanced serum sensitivity of *A. baumannii*. (A) Determination of the optimal volume ratio of enzyme-pretreated bacteria to serum. A total of 10^7^ CFUs of enzyme-pretreated AB1610 were incubated with human serum at a volume ratio of 3:1, 1:1, or 1:3. After samples were incubated for 3 h at 37 °C, the data were recorded as the number of bacterial reduction (initial inoculum minus viable counts). (B) Evaluation of the role of serum complement in serum killing assay. The enzyme-treated bacteria AB1610 were added to active or inactivated serum (56 °C for 30 min), and the original bacteria were incubated with Dpo48 or serum as a control. Data are expressed as the counts of bacterial reduction (means ± SD; *n* = 3). The Student’s *t* test was adopted in two groups comparison, and the one-way ANOVA was conducted to compare multiple groups (**P < 0.01, ***P < 0.001, ****P < 0.0001).

The enzyme-treated host bacteria AB1610 significantly decreased in number when they were mixed with different volumes of serum, as compared with untreated bacteria (Student’s *t* test, *P* < 0.001). The optimal volume ratio of enzyme-treated bacteria to serum was 1:1 (Fig. 5A). In other words, approximately five logs of enzyme-treated bacteria were killed by a 50% volume of serum, and the number of enzyme-treated bacteria did not significantly decrease further, despite the volume of serum being increased from 50% to 75%. For evaluating the role of serum complement in the serum killing assays, the viable counts of enzyme-treated bacteria mixed with active or inactive serum were determined (Fig. 5B). The number of surviving enzyme-pretreated bacteria incubated with active serum effectively decreased as compared with the other three groups (one-way ANOVA, *P* < 0.0001). In contrast, when the enzyme-treated bacteria were incubated with inactive serum, the reduction in the number of bacteria was almost the same as that in two control groups (one-way ANOVA, *P* = 0.2048).

### Homology analysis of phage tail fiber proteins with polysaccharide depolymerase activity

**Figure 6:**
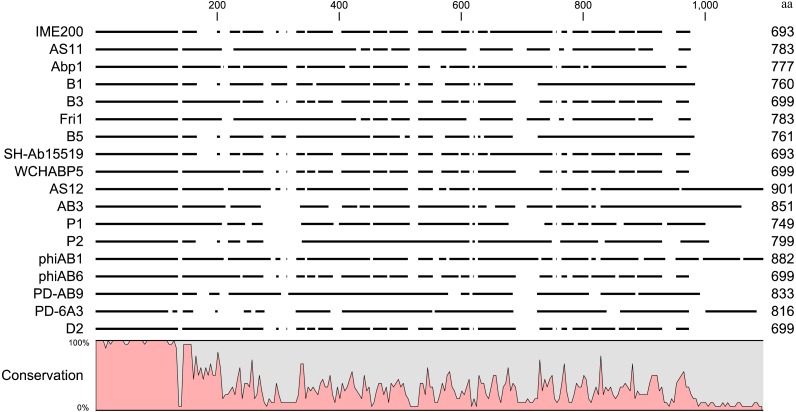
Sequence alignment of putative tail fiber proteins with a polysaccharide depolymerase domain from homologous phages. Amino acid sequence alignment of the tail fiber proteins with confirmed or putative polysaccharide depolymerases encoded by IME200 and its 17 most homologous phages was performed in CLC Genomics Workbench 3.6.1. High and low conservation of amino acid sequences is represented by 100% and 0%, respectively.

Online BLAST analysis showed that the complete genomic sequence of phage IME200 shares high similarity of 93% to 97% with that of 17 *Acinetobacter* phages, viz. AS11 (KY268296.1), Abp1 (JX658790.1), B1 (MF033347.1), B3 (MF033348.1), Fri1 (KR149290.1), B5 (MF033349.1), SH-Ab 15519 (KY082667.1), WCHABP5 (KY888680.2), AS12 (KY268295.1), AB3 (KC311669.1), P1 (MF033350.1), P2 (MF033351.1), phiAB1 (HQ186308.1), phiAB6 (KT339321.1), PD-AB9 (KT388103.1), PD-6A3 (KT388102.1) and D2 (MH042230.1) (Chang et al., 2011; Hua et al., 2017; Huang et al., 2013; Lai et al., 2016; Oliveira et al., 2017; Popova et al., 2017; Zhang, Liu & Li, 2015), all of which belong to the genus *Fri1virus* within the subfamily *Autographivirinae* (Turner et al., 2017). Whole-genomic comparison indicated that these phage genomes have similar structures, but the regions encoding phage tail fiber proteins are significantly different. Because the phage tail fiber gene is related to host specificity and may encode a polysaccharide depolymerase, the amino acid sequences encoded by putative tail fiber proteins were compared. Results of an alignment by the neighbor-joining method indicated that although these phage tail fiber proteins have a relatively short, highly conserved region (from 1 aa–150 aa) at their N-terminal regions, most of their amino acid sequences were obviously diverse in their C-terminal regions (Fig. 6).

## Discussion

Obstacles preventing the application of phages in clinical practice include the fact that a therapy with a phage as a complete virion is hard to standardize for mass production and preservation and poses challenges with prescribing. In addition, whole phage genomes contain some genes of unknown function (Table S2), making it difficult to predict the long-term safety of phages. If some derivatives of phages could be identified and proved to be effective in controlling bacterial infections, this accomplishment may greatly facilitate or accelerate their application to combating multidrug-resistant or extensively drug-resistant *A. baumannii* infections. For Gram-positive bacteria, certain endolysins have been identified as effective antimicrobials *in vitro* and *in vivo* (Chang, Kim & Ryu, 2017; Chang et al., 2017; Zhou et al., 2017). In Gram-negative bacteria, the thick capsule surrounding the cell wall protects the bacteria from peptidoglycan-degrading enzymes; therefore, few endolysins themselves can permeate the cell membrane for an antibacterial effect. There are recent reports that some endolysins have a broad anti Gram-negative bacterial activity (Dong et al., 2015; Guo et al., 2017a; Oliveira et al., 2014; Oliveira et al., 2016; Thummeepak et al., 2016). Nonetheless, most of the endolysins were found to have an elevated antibacterial activity in the presence of outer-membrane permeabilizers (Oliveira et al., 2014; Oliveira et al., 2016). Fortunately, most of the polysaccharide depolymerases commonly encoded by phage tail fiber genes have been found to have antivirulence functions by destroying the bacterial capsule (Lin et al., 2014b; Lin et al., 2017).

It is known that the phage tail fiber or tail spike protein with a Pectate_lyase_3 domain often functions as a polysaccharide depolymerase (Pires et al., 2016). Our study confirmed that the identified tail fiber protein with a Pectate_lyase_3 domain encoded by ORF48 has a depolymerase activity. Although most depolymerases are encoded by tail fiber or tail spike genes, they are sometimes located in the phage structure of the base plates, in the preneck appendage genes, or in close proximity to them (Gutierrez et al., 2015; Pires et al., 2016). According to previous research, the majority of depolymerases belong to the class of pectolytic enzymes and contain predicted Pectate_lyase_3 domains, in agreement with ORF48 in our study. Nonetheless, besides the domain of Pectate_lyase_3, the phage proteins with a Pec_lyase_C domain or a Pectin lyase-like domain also have pectolytic enzyme activity (Pires et al., 2016). As previously reported, pectolytic enzymes can normally degrade polygalacturonic acid, which is a major component of bacterial surface polysaccharides (Garron & Cygler, 2010). Our results indicate that Dpo48 degrades the bacterial CPS as described in other reports (Hsieh et al., 2017; Karlyshev & Wren, 2001; Pan et al., 2013), according to Alcian blue staining. Nevertheless, some pectolytic enzymes were shown to degrade bacterial lipopolysaccharide (LPS) (Lee et al., 2017).

Our results indicate that Dpo48 has stable activity under moderate acidic or alkaline conditions (pH 5.0–9.0), with an optimal pH of 5.0. Nevertheless, the optimal pH of enzymes from the phage targeted to *Klebsiella pneumoniae* B5055 is 7.5, and the enzymatic activity is stable under relatively alkaline conditions (pH 7.0–9.0) (Kassa & Chhibber, 2012). The optimal temperature for enzymes from phages specific to *K. pneumoniae* B5055 and *Klebsiella* KP36 is 40 °C and 20–37 °C, respectively (Kassa & Chhibber, 2012; Majkowska-Skrobek et al., 2016). However, Dpo48 activity was optimal at temperatures ranging from 20 to 70 °C. Therefore, Dpo48 ensures high efficacy in a relatively broad range of pH and temperatures.

Depolymerase can strip bacterial capsules and thus sensitize bacteria to killing by serum (Lin et al., 2014b; Lin et al., 2017). To determine whether Dpo48 could sensitize the *A. baumannii* strains to serum, the bacterial reduction of the enzyme-pretreated bacteria mixed with serum was analyzed. In some studies, most of approximately 10^4^ CFUs of enzyme-treated log phase bacteria could be killed by a 75% volume of serum (Hsieh et al., 2017; Lin et al., 2014b; Lin et al., 2017; Pan et al., 2015); these data are consistent with our findings (Fig. S1). Nevertheless, some log-phase bacteria are highly sensitive to serum (Bruhn et al., 2015; Harris et al., 2013; Lin et al., 2014a; Siu et al., 2011); therefore, the overnight-cultured bacteria, which have thicker bacterial capsules, were used to evaluate the antivirulence abilities of Dpo48 by us. Besides, approximately 10^7^ CFUs of enzyme-pretreated bacteria were used for examining the effects of serum on higher doses of bacteria. Our results indicated that approximately five logs of the enzyme-pretreated overnight-cultured bacteria were killed by a 50% volume of serum. Therefore, this enzyme can be considered as an antivirulence agent for prevention of *A. baumannii* infections *in vivo*. Moreover, our data suggest that serum complement plays an important role in serum killing assays, in line with other findings (Lin et al., 2017).

Our previous research showed that when treated with lytic bacteriophages, some host bacteria can develop resistance and escape eradication by lytic phages (Liu et al., 2016). In this study, we found that the number of enzyme-pretreated bacteria is not significantly decreased further when the ratio of serum volume is increased. Some studies suggest that 75% of bacteria are killed after phage treatment (multiplicity of infection, 1000) for 30 min, while the survivors are all nonencapsulated (Lin et al., 2014b). Our results indicated that even a high dose of Dpo48 (2 µg) failed to yield translucent halos on the lawn of enzyme-treated bacteria, as revealed by modified single-spot assays, suggesting that all enzyme-treated bacteria may lack capsules (Fig. S2). Therefore, we speculated that although all *A. baumannii* cells originate from a single clone, selection pressure by serum causes retention of some cells with serum-resistant phenotypes during the growth period, thereby preventing their disruption by serum complement.

It is well known that phages normally infect specific bacteria. Results of our modified single-spot assays imply that Dpo48 strips bacterial capsules and acts against the same strains infected by the intact phage IME200 (Table 2). This similarity can be explained by recent findings of Oliveira et al. (2017), who showed that bacterial capsules are the primary phage receptor, whereas the pectate lyase domains related to polysaccharide depolymerase activity are responsible for binding to specific *A. baumannii* bacterial capsules. Furthermore, comparisons based on amino acid sequences of tail fiber genes confirmed that the C-terminal sequences containing pectate lyase domains were significantly different here; this finding may explain why the phage and its depolymerase have such high specificity to certain bacteria. If the tail fiber genes are replaced with those from *Acinetobacter* phages, which encode pectate lyase domains, then the phage can lyse new hosts and is not sensitive to the original hosts (Lai et al., 2016). Therefore, we need to identify and characterize more polysaccharide depolymerases to target different capsule types of *A. baumannii* for enhancing the antivirulence activity.

## Conclusions

Capsule depolymerase Dpo48 has a relatively broad activity spectrum and maintains stable activity in a broad range of pH and temperatures. At a high concentration of overnight-cultured *A. baumannii*, capsules were quickly stripped by minimal concentrations of Dpo48, and the bacteria were next killed by serum complement *in vitro*. Overall, phage-derived depolymerases may be promising antivirulence agents for preventing multidrug-resistant or extensively drug-resistant *A. baumannii* infections.

##  Supplemental Information

10.7717/peerj.6173/supp-1 Table S1 The antibiotic resistance profile of the clinical strain *A. baumannii* AB1610

10.7717/peerj.6173/supp-2 Table S2 The list of ORFs in the genome of phage IME200 and their putative functions

10.7717/peerj.6173/supp-3 Data S1 Raw data

10.7717/peerj.6173/supp-4 Figure S1 Dpo48 enhanced serum sensitivity of *A. baumannii* A total of 10^4^ CFUs of enzyme-treated AB1610 were incubated with human serum at a volume ratio of 1:3. After 3 h at 37 °C, the data were recorded as the survival ratio of bacteria (CFUs of viable bacteria /CFUs of initial inoculum). Data are expressed as the survival ratio of bacteria (means ± SD; *n* = 3), and Student’s *t* test was carried out to compare the groups (****P < 0.0001).

10.7717/peerj.6173/supp-5 Figure S2 Enzyme-treated *A. baumannii* was not sensitive to Dpo48 The sensitivity of the host (A) or enzyme-treated bacterium AB1610 (B) to Dpo48 (2 *μ*g) was determined by modified single-spot assays. Formation of a transparent halo as a measure of bacterial sensitivity. PBS served as a negative control.

## Additional Information and Declarations

### DNA Deposition

The following information was supplied regarding the deposition of DNA sequences: The sequence of bacteriophage IME200 described here are accessible via GenBank accession number KT804908.2.

### Data Availability

The following information was supplied regarding data availability: The raw data has been supplied as a Supplemental File.
