# Supplementary material for: Identification and characterization of capsule depolymerase Dpo48 from Acinetobacter baumannii phage IME200
Source: PeerJ. 2019 Jan 14;7:e6173. doi: 10.7717/peerj.6173 (PMC6336015; doi:10.7717/peerj.6173)
Supplement: Table S1 [file peerj-07-6173-s001.doc]

**Supplementary Table 1. The antibiotic resistance profile of the clinical strain *A. baumannii* AB1610**

| **Antimicrobial category** | **Antimicrobial agent** | **MIC (μg/mL)** | **Sensitivity** |
| --- | --- | --- | --- |
| Aminoglycosides | Amikacin | ≥ 64 | Resistant |
|  | Gentamicin | ≥ 16 | Resistant |
|  | Tobramycin | ≥ 16 | Resistant |
| Antipseudomonal carbapenems | Imipenem | ≥ 8 | Resistant |
|  | Meropenem | ≥ 8 | Resistant |
| Antipseudomonal fluoroquinolones | Ciprofloxacin | ≥ 4 | Resistant |
|  | Levofloxacin | ≥ 8 | Resistant |
| Antipseudomonal penicillins  + *β*-lactamase inhibitors | Piperacillin-tazobactam | ≥ 128 | Resistant |
|  | Ticarcillin-clavulanic acid | ≥ 128 | Resistant |
| Extended-spectrum cephalosporins | Ceftriaxone | ≥ 64 | Resistant |
|  | Ceftazidime | ≥ 64 | Resistant |
|  | Cefepime | ≥ 64 | Resistant |
| Folate pathway inhibitors | Trimethoprim-sulphamethoxazole | ≥ 320 | Resistant |
| Penicillins + *β*-lactamase inhibitors | Ampicillin-sulbactam | ≥ 32 | Resistant |
| Polymyxins | Polymyxin B | ≥ 4 | Resistant |
| Tetracyclines | Doxycycline | ≥ 16 | Resistant |
|  | Minocycline | ≥ 32 | Resistant |
|  | Tigecycline | ≥ 8 | Resistant |

MIC: minimum inhibitory concentration
