# Supplementary material for: Identification and characterization of capsule depolymerase Dpo48 from Acinetobacter baumannii phage IME200
Source: PeerJ. 2019 Jan 14;7:e6173. doi: 10.7717/peerj.6173 (PMC6336015; doi:10.7717/peerj.6173)
Supplement: Table S2 [file peerj-07-6173-s002.docx]

**Supplementary Table 2. The list of ORFs in the genome of phage IME200 and their putative functions.**

| **ORF** | **Location (nt)^a^** | **Product**  **Length (aa)^b^** | **Homologue** | **Accession number** | **Sequence**  **identity (%)^c^** |
| --- | --- | --- | --- | --- | --- |
| 1 | 916..1122 | 68 | hypothetical protein of Acinetobacter phage Abp1 | YP_008058193.1 | 97 |
| 2 | 1554..2024 | 156 | hypothetical protein of Acinetobacter phage IME200 | YP_009216497.1 | 100 |
| 3 | 2094..2603 | 169 | hypothetical protein of Acinetobacter phage vB_AbaP_PD-AB9 | YP_009189871.1 | 100 |
| 4 | 2605..2979 | 124 | hypothetical protein of Acinetobacter phage Fri1 | YP_009203012.1 | 90 |
| 5 | 2970..3083 | 37 | hypothetical protein of Acinetobacter phage vB_AbaP_D2 | AVP40482.1 | 97 |
| 6 | 3070..3303 | 77 | hypothetical protein of Acinetobacter phage vB_AbaP_PD-AB9 | YP_009189868.1 | 96 |
| 7 | 3378..3974 | 198 | hypothetical protein of Acinetobacter phage phiAB6 | YP_009288635.1 | 98 |
| 8 | 3971..4150 | 59 | hypothetical protein of Acinetobacter phage Abp1 | YP_008058199.1 | 86 |
| 9 | 4131..4247 | 38 | hypothetical protein of Acinetobacter phage Abp1 | YP_008058200.1 | 92 |
| 10 | 4234..4395 | 53 | hypothetical protein of Acinetobacter phage Fri1 | YP_009203016.1 | 92 |
| 11 | 4405..4803 | 132 | hypothetical protein of Acinetobacter phage vB_ApiP_P2 | ASN73521.1 | 98 |
| 12 | 4881..5381 | 166 | hypothetical protein of Acinetobacter phage vB_AbaP_AS12 | APW79796.1 | 90 |
| 13 | 5383..5820 | 145 | hypothetical protein of Acinetobacter phage vB_AbaP_AS12 | APW79797.1 | 95 |
| 14 | 5831..5998 | 55 | hypothetical protein of Acinetobacter phage vB_ApiP_P1 | ASN73474.1 | 98 |
| 15 | 5985..6176 | 63 | hypothetical protein of Acinetobacter phage WCHABP5 | ARQ94884.1 | 94 |
| 16 | 6173..6391 | 72 | hypothetical protein of Acinetobacter phage phiAB1 | YP_009189349.1 | 94 |
| 17 | 6381..6605 | 74 | hypothetical protein of Acinetobacter phage vB_ApiP_P1 | ASN73476.1 | 80 |
| 18 | 6595..7395 | 266 | putative DNA primase of Acinetobacter phage phiAB1 | YP_009189351.1 | 98 |
| 19 | 7395..7712 | 105 | hypothetical protein of Acinetobacter phage phiAB1 | YP_009189352.1 | 96 |
| 20 | 7712..7954 | 80 | hypothetical protein of Acinetobacter phage Fri1 | YP_009203026.1 | 76 |
| 21 | 7967..9265 | 432 | DNA helicase of Acinetobacter phage vB_AbaP_B5 | ASN73430.1 | 99 |
| 22 | 9268..10005 | 245 | hypothetical protein of Acinetobacter phage vB_AbaP_AS11 | AQN32671.1 | 93 |
| 23 | 10002..10979 | 325 | putative ATP-dependent DNA ligase of Acinetobacter phage SH-Ab 15519 | APD19412.1 | 96 |
| 24 | 10972..11055 | 27 | hypothetical protein of Acinetobacter phage phiAB6 | YP_009288649.1 | 93 |
| 25 | 11228..13525 | 765 | DNA polymerase I of Acinetobacter phage vB_AbaP_AS12 | APW79807.1 | 94 |
| 26 | 13534..13956 | 140 | HNH homing endonuclease of Acinetobacter phage vB_AbaP_AS12 | APW79808.1 | 99 |
| 27 | 13959..14849 | 296 | hypothetical protein of Acinetobacter phage vB_AbaP_B1 | ASN73332.1 | 98 |
| 28 | 14906..15025 | 39 | hypothetical protein of Acinetobacter phage vB_ApiP_P2 | ASN73538.1 | 97 |
| 29 | 15022..15354 | 110 | hypothetical protein of Acinetobacter phage vB_AbaP_B1 | ASN73334.1 | 96 |
| 30 | 15347..16300 | 317 | 5'-3' exonuclease of Acinetobacter phage vB_AbaP_AS12 | APW79812.1 | 99 |
| 31 | 16290..16859 | 188 | hypothetical protein of Acinetobacter phage IME200 | YP_008060152.1 | 100 |
| 32 | 16856..17296 | 146 | putative DNA endonuclease VII of Acinetobacter phage phiAB1 | YP_009189364.1 | 99 |
| 33 | 17300..18235 | 311 | hypothetical protein of Acinetobacter phage phiAB1 | YP_009189365.1 | 100 |
| 34 | 18235..18885 | 216 | dNMP kinase of Acinetobacter phage vB_AbaP_AS12 | APW79816.1 | 98 |
| 35 | 18894..21311 | 805 | RNA polymerase of Acinetobacter phage vB_AbaP_D2 | AVP40508.1 | 99 |
| 36 | 21417..21614 | 65 | hypothetical protein of Acinetobacter phage vB_AbaP_B3 | ASN73389.1 | 98 |
| 37 | 21611..21862 | 83 | hypothetical protein of Acinetobacter phage vB_AbaP_B1 | ASN73342.1 | 99 |
| 38 | 21871..23427 | 518 | head-tail connector protein of Acinetobacter phage vB_AbaP_AS11 | AQN32687.1 | 99 |
| 39 | 23436..24296 | 286 | scaffolding protein of Acinetobacter phage vB_AbaP_B5 | ASN73446.1 | 97 |
| 40 | 24312..25343 | 343 | putative capsid protein of Acinetobacter phage phiAB1 | YP_009189372.1 | 99 |
| 41 | 25397..25582 | 61 | hypothetical protein of Acinetobacter phage AB3 | YP_008060142.1 | 98 |
| 42 | 25594..25887 | 97 | hypothetical protein of Acinetobacter phage WCHABP5 | ARQ94910.1 | 98 |
| 43 | 26028..26588 | 186 | tail tubular protein A of Acinetobacter phage AB3 | YP_008060141.1 | 99 |
| 44 | 26597..28888 | 763 | tail tubular protein B of Acinetobacter phage vB_AbaP_B3 | ASN73397.1 | 94 |
| 45 | 28888..29559 | 223 | hypothetical protein of Acinetobacter phage Abp1 | YP_008058236.1 | 99 |
| 46 | 29572..32457 | 961 | hypothetical protein of Acinetobacter phage vB_AbaP_PD-AB9 | YP_009189832.1 | 98 |
| 47 | 32467..35565 | 1032 | putative internal virion protein C of Acinetobacter phage Fri1 | YP_009203054.1 | 99 |
| 48 | 35572..37653 | 693 | putative tail fiber protein of Acinetobacter phage SH-Ab 15519 | APD19440.1 | 97 |
| 49 | 37666..38001 | 111 | putative holin of Acinetobacter phage SH-Ab 15519 | APD19439.1 | 99 |
| 50 | 37988..38545 | 185 | endolysin of Acinetobacter phage vB_AbaP_B3 | ASN73403.1 | 98 |
| 51 | 38606..38914 | 102 | putative DNA maturase A of Acinetobacter phage Fri1 | YP_009203058.1 | 97 |
| 52 | 38924..40861 | 645 | putative DNA maturase B of Acinetobacter phage phiAB6 | YP_009288675.1 | 99 |
| 53 | 40858..40995 | 45 | hypothetical protein Abp1_0053 of Acinetobacter phage Abp1 | YP_008058245.1 | 100 |
| 54 | 40952..41155 | 67 | hypothetical protein of Acinetobacter phage vB_AbaP_AS11 | AQN32703.1 | 97 |

^a^ nt, nucleotide; ^b^ aa, amino acid; ^c^ determined by BLAST-P.
