# Supplementary material for: Identification and characterization of capsule depolymerase Dpo48 from Acinetobacter baumannii phage IME200
Source: PeerJ. 2019 Jan 14;7:e6173. doi: 10.7717/peerj.6173 (PMC6336015; doi:10.7717/peerj.6173)
Supplement: Data S1 [file peerj-07-6173-s003.docx]

**Table 1. Susceptibility rates toward different classes of antibiotics for clinical strains of *A. baumannii*.**

| **Antimicrobial category** | **Antimicrobial agent** | **Bacteria** | | | | | | | | | |
| --- | --- | --- | --- | --- | --- | --- | --- | --- | --- | --- | --- |
|  |  | **AB1** | **AB2** | **AB3** | **AB4** | **AB5** | **AB6** | **AB7** | **AB8** | **AB9** | **AB10** |
| Penicillins + β-lactamase inhibitors | Ampicillin-sulbactam | I | R | R | R | R | R | R | R | R | R |
| Antipseudomonal penicillins  +β-lactamase inhibitors | Piperacillin-tazobactam | R | R | R | R | R | R | R | R | R | R |
| Extended-spectrum cephalosporins | Cefepime | R | R | R | R | R | R | R | R | R | R |
|  | Ceftazidime | R | R | R | R | R | R | R | R | R | R |
|  | Ceftriaxone | R | R | R | R | R | R | R | R | R | R |
| Antipseudomonal carbapenems | Imipenem | R | R | R | R | R | R | R | R | R | R |
|  | Meropenem | R | R | R | R | R | R | R | R | R | R |
| Aminoglycosides | Amikacin | R | R | R | R | R | S | R | S | S | S |
|  | Gentamicin | R | R | R | R | R | R | R | R | R | R |
|  | Tobramycin | R | R | R | R | R | R | R | R | R | R |
| Folate pathway inhibitors | Trimethoprim-sulphamethoxazole | R | R | R | R | R | S | S | R | R | R |
| Antipseudomonal fluoroquinolones | Ciprofloxacin | R | R | R | R | R | R | R | R | R | R |
|  | Levofloxacin | R | R | R | R | R | R | I | R | R | R |

Note: S, Susceptible; I, Intermediate; R, Resistant.

**Table 1. Susceptibility rates toward different classes of antibiotics for clinical strains of *A. baumannii*.**

| **Antimicrobial category** | **Antimicrobial agent** | **Bacteria** | | | | | | | | | |
| --- | --- | --- | --- | --- | --- | --- | --- | --- | --- | --- | --- |
|  |  | **AB11** | **AB12** | **AB13** | **AB14** | **AB15** | **AB16** | **AB17** | **AB18** | **AB19** | **AB20** |
| Penicillins + β-lactamase inhibitors | Ampicillin-sulbactam | R | R | R | R | I | R | I | R | S | R |
| Antipseudomonal penicillins  +β-lactamase inhibitors | Piperacillin-tazobactam | R | I | R | R | R | R | R | R | S | R |
| Extended-spectrum cephalosporins | Cefepime | R | R | R | R | R | R | R | R | S | R |
|  | Ceftazidime | R | R | R | R | I | I | I | R | I | R |
|  | Ceftriaxone | R | R | R | R | R | I | R | R | I | R |
| Antipseudomonal carbapenems | Imipenem | R | S | R | R | R | R | R | R | S | R |
|  | Meropenem | R | S | R | R | R | R | R | R | S | R |
| Aminoglycosides | Amikacin | R | S | R | S | S | S | R | S | S | S |
|  | Gentamicin | R | R | R | R | S | R | R | R | S | S |
|  | Tobramycin | R | R | R | R | S | R | R | R | S | S |
| Folate pathway inhibitors | Trimethoprim-sulphamethoxazole | S | S | R | R | R | S | R | R | S | R |
| Antipseudomonal fluoroquinolones | Ciprofloxacin | R | R | R | R | R | R | R | R | S | R |
|  | Levofloxacin | R | I | R | I | R | R | R | R | S | R |

Note: S, Susceptible; I, Intermediate; R, Resistant.

**Table 1. Susceptibility rates toward different classes of antibiotics for clinical strains of *A. baumannii*.**

| **Antimicrobial category** | **Antimicrobial agent** | **Bacteria** | | | | | | | | | |
| --- | --- | --- | --- | --- | --- | --- | --- | --- | --- | --- | --- |
|  |  | **AB007** | **AB043** | **AB139** | **AB178** | **AB220** | **AB237** | **AB295** | **AB358** | **AB363** | **AB387** |
| Penicillins + β-lactamase inhibitors | Ampicillin-sulbactam | R | R | R | R | S | S | S | R | R | R |
| Antipseudomonal penicillins  +β-lactamase inhibitors | Piperacillin-tazobactam | I | R | R | R | S | S | S | R | R | I |
| Extended-spectrum cephalosporins | Cefepime | R | R | R | R | S | S | S | R | R | R |
|  | Ceftazidime | R | R | R | R | S | R | S | R | R | R |
|  | Ceftriaxone | R | R | R | R | I | I | I | R | R | R |
| Antipseudomonal carbapenems | Imipenem | R | R | R | R | S | S | S | R | R | R |
|  | Meropenem | R | R | R | R | S | S | S | R | R | R |
| Aminoglycosides | Amikacin | I | S | S | I | S | S | S | S | S | S |
|  | Gentamicin | R | R | R | R | S | S | S | R | R | R |
|  | Tobramycin | R | R | R | R | S | S | S | R | R | R |
| Folate pathway inhibitors | Trimethoprim-sulphamethoxazole | R | R | R | R | S | S | S | S | S | R |
| Antipseudomonal fluoroquinolones | Ciprofloxacin | R | R | R | R | S | S | S | R | R | R |
|  | Levofloxacin | I | R | R | I | S | S | S | I | R | I |

Note: S, Susceptible; I, Intermediate; R, Resistant.

**Table 1. Susceptibility rates toward different classes of antibiotics for clinical strains of *A. baumannii*.**

| **Antimicrobial category** | **Antimicrobial agent** | **Bacteria** | | | | | | | | | | |
| --- | --- | --- | --- | --- | --- | --- | --- | --- | --- | --- | --- | --- |
|  |  | **AB392** | **AB406** | **AB1610** | **AB1611** | **AB1612** | **AB1613** | **AB1614** | **AB1615** | **AB5-2** | **AB-E85** | **L-AB9** |
| Penicillins + β-lactamase inhibitors | Ampicillin-sulbactam | R | R | R | R | S | R | R | R | R | R | S |
| Antipseudomonal penicillins  +β-lactamase inhibitors | Piperacillin-tazobactam | R | I | R | R | S | I | R | R | R | R | S |
| Extended-spectrum cephalosporins | Cefepime | R | R | R | R | I | I | R | R | R | R | S |
|  | Ceftazidime | R | R | R | R | I | I | R | R | R | R | R |
|  | Ceftriaxone | R | R | R | R | R | R | R | R | R | R | I |
| Antipseudomonal carbapenems | Imipenem | R | R | R | R | S | R | R | R | R | R | S |
|  | Meropenem | R | R | R | R | S | R | R | R | R | R | S |
| Aminoglycosides | Amikacin | R | S | R | S | S | S | S | S | R | R | S |
|  | Gentamicin | R | R | R | R | S | R | R | R | R | R | S |
|  | Tobramycin | R | R | R | R | S | S | R | R | R | R | S |
| Folate pathway inhibitors | Trimethoprim-sulphamethoxazole | S | R | R | R | R | R | R | S | S | R | S |
| Antipseudomonal fluoroquinolones | Ciprofloxacin | R | R | R | R | R | R | R | R | R | R | S |
|  | Levofloxacin | R | I | R | R | R | I | R | R | R | R | S |

Note: S, Susceptible; I, Intermediate; R, Resistant.

**Figure 3A. Bacterial surface polysaccharides were degraded by Dpo48.**

|  | Different treatments | | | |
| --- | --- | --- | --- | --- |
|  | Untreated EPS | EPS + Inactive Dpo48 | EPS + Active Dpo48 | Dpo48 |
| OD_540_ values | 0.121 | 0.134 | 0.458 | 0.123 |
|  | 0.128 | 0.125 | 0.393 | 0.126 |
|  | 0.117 | 0.128 | 0.405 | 0.129 |

**Figure 4A. The influence of pH on the activity of Dpo48.**

|  | Control | | pH | | | | | | |
| --- | --- | --- | --- | --- | --- | --- | --- | --- | --- |
|  | EPS | Dpo48 | 3 | 4 | 5 | 6 | 7 | 8 | 9 |
| OD_540_  Values | 0.125 | 0.128 | 0.114 | 0.278 | 0.477 | 0.389 | 0.401 | 0.375 | 0.39 |
|  | 0.126 | 0.129 | 0.125 | 0.31 | 0.523 | 0.419 | 0.429 | 0.402 | 0.417 |
|  | 0.131 | 0.129 | 0.134 | 0.324 | 0.548 | 0.441 | 0.44 | 0.415 | 0.427 |
|  | 0.126 | 0.127 | 0.137 | 0.317 | 0.519 | 0.432 | 0.433 | 0.412 | 0.415 |

**Figure 4B. The effect of temperature on Dpo48 activity.**

|  | Control | | Temperatures | | | | | |
| --- | --- | --- | --- | --- | --- | --- | --- | --- |
|  | EPS | Dpo48 | 20 | 37 | 50 | 60 | 70 | 80 |
| OD_540_  Values | 0.11 | 0.112 | 0.47 | 0.45 | 0.453 | 0.4 | 0.422 | 0.338 |
|  | 0.108 | 0.109 | 0.538 | 0.498 | 0.499 | 0.472 | 0.457 | 0.374 |
|  | 0.111 | 0.108 | 0.554 | 0.524 | 0.516 | 0.495 | 0.48 | 0.417 |
|  | 0.112 | 0.116 | 0.563 | 0.561 | 0.508 | 0.503 | 0.469 | 0.375 |

**Figure 5A. Determination of the optimal volume ratio of enzyme-pretreated bacteria to serum.**

|  | Dpo48 (Bacterial reduction) | | | Control (Bacterial reduction) | | |
| --- | --- | --- | --- | --- | --- | --- |
| 75 % Serum | 5.051152522 | 5.102662000 | 4.720159303 | 0.808114474 | 0.831595570 | 0.419129308 |
| 50 % Serum | 4.750122527 | 4.898542000 | 4.720159303 | 0.282144652 | 0.530565574 | 0.208275942 |
| 25 % Serum | 3.213879820 | 3.831596000 | 3.118099312 | -0.124938737 | -0.146128036 | -0.039508541 |

**Figure 5B. Evaluation of the role of serum complement in serum killing assay.**

|  | Different treatments | | | |
| --- | --- | --- | --- | --- |
|  | Dpo48 | Serum | Dpo48 + Serum | Dpo48 + Inactive Serum |
| Bacterial reduction | 0.168912674 | 0.462880816 | 5.040428657 | 0.522878745 |
|  | -0.063669080 | 0.515325607 | 5.403692338 | 0.676693610 |
|  | -0.441208699 | 0.118099312 | 4.720159303 | -0.124938737 |

**Supplementary Figure 1. Dpo48 enhanced serum sensitivity of *A. baumannii.***

|  | 75 % Serum | | | 75 % Serum + Dpo48 | | |
| --- | --- | --- | --- | --- | --- | --- |
| Survival Ratio | 0.635037148 | 0.731614637 | 0.739543118 | 0.060762063 | 0.118534851 | 0.060475434 |
