# Supplementary figures and images for: Identification and characterization of capsule depolymerase Dpo48 from Acinetobacter baumannii phage IME200
Source: PeerJ. 2019 Jan 14;7:e6173. doi: 10.7717/peerj.6173 (PMC6336015; doi:10.7717/peerj.6173)

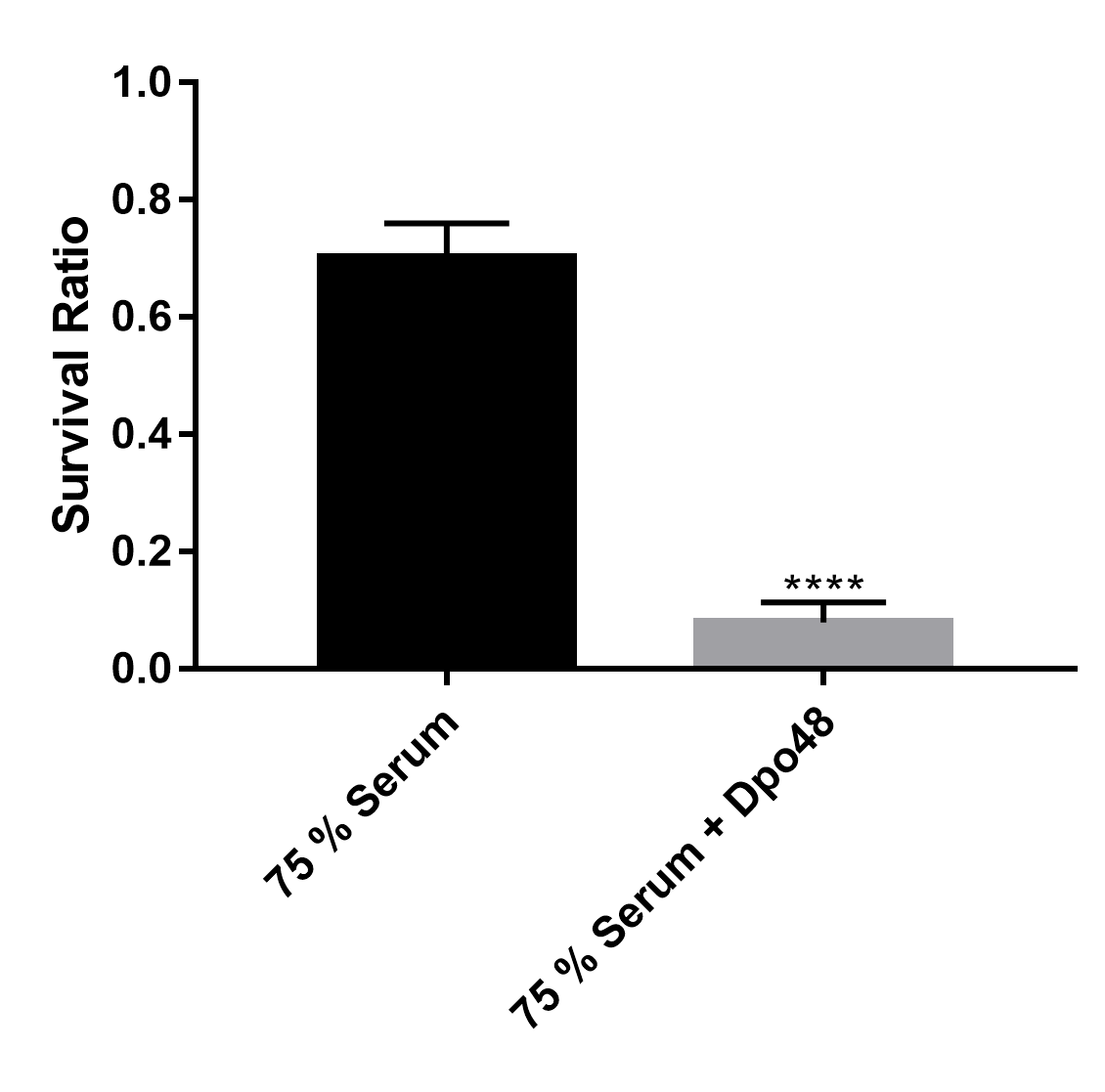

Supplement: Figure S1 — A total of 104 CFUs of enzyme-treated AB1610 were incubated with human serum at a volume ratio of 1:3. After 3 h at 37 °C, the data were recorded as the survival ratio of bacteria (CFUs of viable bacteria /CFUs of initial inoculum). Data are expressed as the survival ratio of bacteria (means ± SD; n = 3), and Student’s t test was carried out to compare the groups (****P < 0.0001). [file peerj-07-6173-s004.png]

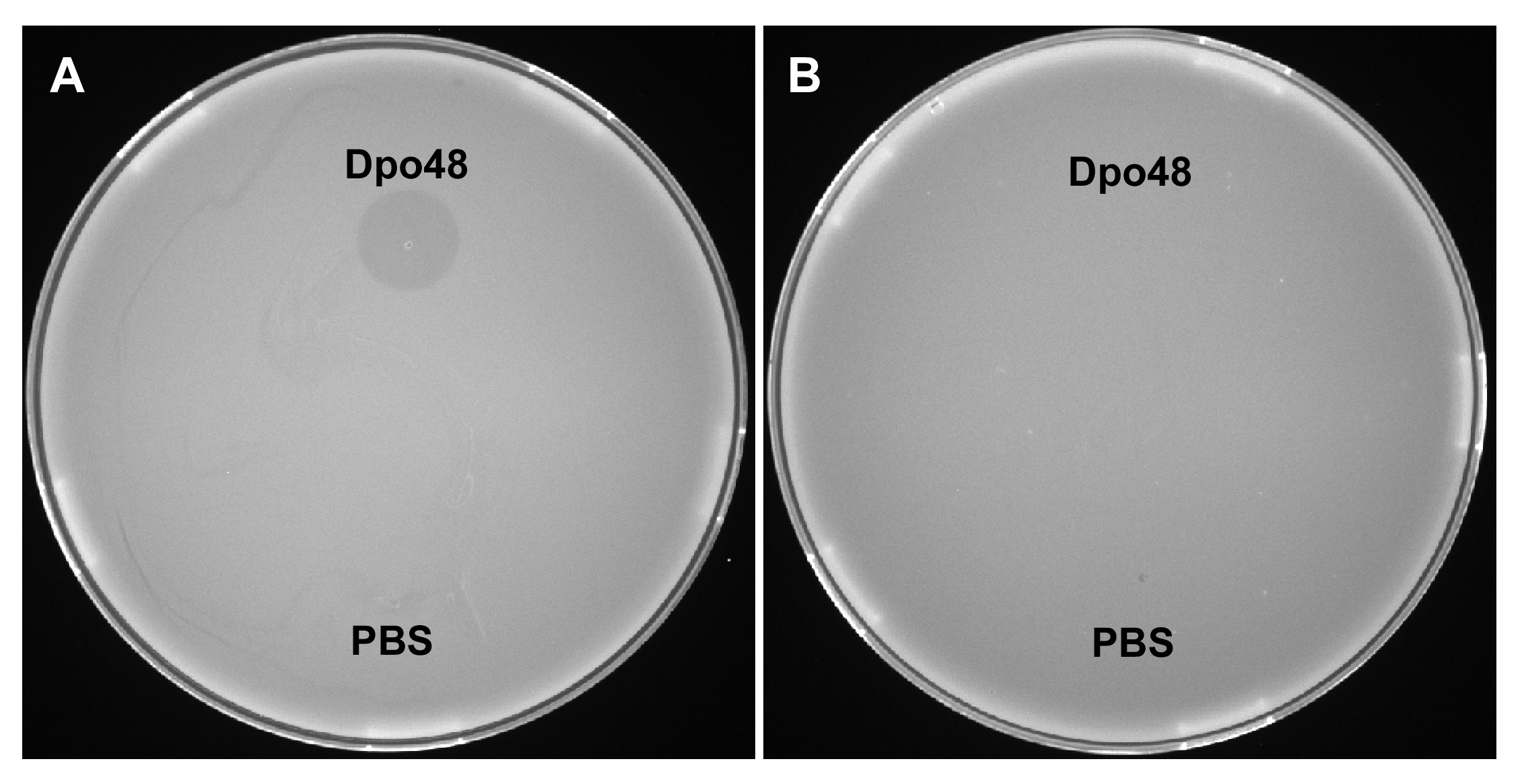

Supplement: Figure S2 — The sensitivity of the host (A) or enzyme-treated bacterium AB1610 (B) to Dpo48 (2 μg) was determined by modified single-spot assays. Formation of a transparent halo as a measure of bacterial sensitivity. PBS served as a negative control. [file peerj-07-6173-s005.png]
